# West Nile Virus Economic Impact, Louisiana, 2002

**DOI:** 10.3201/eid1010.030925

**Published:** 2004-10

**Authors:** Armineh Zohrabian, Martin I. Meltzer, Raoult Ratard, Kaafee Billah, Noelle A. Molinari, Kakoli Roy, R. Douglas Scott, Lyle R. Petersen

**Affiliations:** * Centers for Disease Control and Prevention, Atlanta, Georgia, USA;; † Louisiana Department of Health and Hospitals, New Orleans, Louisiana, USA

**Keywords:** West Nile virus, cost of illness, hospital costs, disease outbreaks, perspective

## Abstract

West Nile virus (WNV) is transmitted by mosquitoes and can cause illness in humans ranging from mild fever to encephalitis. In 2002, a total of 4,156 WNV cases were reported in the United States; 329 were in Louisiana. To estimate the economic impact of the 2002 WNV epidemic in Louisiana, we collected data from hospitals, a patient questionnaire, and public offices. Hospital charges were converted to economic costs by using Medicare cost-to-charge ratios. The estimated cost of the Louisiana epidemic was $20.1 million from June 2002 to February 2003, including a $10.9 million cost of illness ($4.4 million medical and $6.5 million nonmedical costs) and a $9.2 million cost of public health response. These data indicate a substantial short-term cost of the WNV disease epidemic in Louisiana.

## Abstract 2

2002 WNV epidemic in Louisiana incurred substantial short-term economic costs

West Nile virus (WNV) is transmitted by mosquitoes and can cause illnesses ranging from simple fevers to encephalitis (*1*). The presence of this virus in the Western Hemisphere was first recognized in New York City in 1999 (*2*). In 2002, an epidemic of WNV illness focused in the midwestern United States resulted in 4,156 reported cases; 2,942 cases had central nervous system (CNS) illness (meningitis, encephalitis, or acute flaccid paralysis), and 284 died (*3*). A total of 329 persons with WNV disease were reported in Louisiana, with illness onsets from June to November. Among these, 204 had illnesses involving the CNS; 24 died (Louisiana Office of Public Health, unpub. data).

Economic data about epidemics are essential for estimating the costs and benefits of strengthening and maintaining prevention and control programs, improving existing surveillance systems, and introducing other proposed interventions, such as vaccines. Although some estimates exist of the economic impact imposed by diseases transmitted by mosquitoes (*4**–**9*), to our knowledge, no previous studies have assessed the costs of a WNV disease epidemic. We estimated the magnitude of the short-term economic costs of the 2002 WNV epidemic in Louisiana.

## Economic Model, Data, and Methods

We calculated the costs of the WNV epidemic as the sum of 1) medical costs (inpatient and outpatient); 2) nonmedical costs, such as productivity losses caused by illness and premature death, costs of transportation for a patient to visit a healthcare provider, and childcare expenses; and 3) costs incurred by public health and other government agencies for epidemic control. Data were gathered from hospitals in Louisiana that had WNV patients; a phone survey of WNV patients (all adult patients with nonfatal cases for whom phone numbers were available from the Louisiana Office of Public Health were included in the survey); and public offices, including the Louisiana Office of Public Health, state and local governments, and the Louisiana Office of Emergency Preparedness. Because information could not be gathered for all hospitalized patients and the patient questionnaire could not be administered to all reported patients, we extrapolated cost data, assuming that the costs for those with information were representative of those without information (the extrapolation method is described in Appendix 1).

We took a societal perspective, evaluating all costs regardless of who bore them. The costs were estimated from June 2002, when the epidemic was first recognized, until the last date we administered the phone survey, February 27, 2003, some 3 months after the onset of illness of the last reported patient. Intangible costs, attributable to factors such as pain and suffering, were not included.

### Medical Costs

#### Inpatient Costs for Acute Care and Rehabilitation

In fall 2002 we requested information from Louisiana hospitals on the length of hospital stay and inpatient and outpatient treatment charges, including therapies at inpatient rehabilitation facilities for patients who met the case definition of probable or confirmed WNV illness (http://www.cdc.gov/epo/dphsi/casedef/encephalitiscurrent.htm). To ensure patient anonymity, patient information from hospitals was given to the study investigators unlinked to personal identifiers, and only the 16 hospitals with more than three adult patients (>18 years old) were queried. Adults constituted 94% of reported WNV case-patients in Louisiana.

Twelve hospitals submitted information from 159 patients, including inpatient treatment charges for 119 patients and hospital outpatient treatment charges for 50 patients. Ten of these 50 patients had both inpatient and outpatient treatment charges. Patient charges included 65 inpatient treatment or service types, which we grouped for the analysis into eight categories (Table 1). For example, we pooled hospital charges originally listed as "pharmacy," "drugs," "injection," "medical/surgical supplies," "IV solutions," "IV therapy," and "prosthetic devices" into the category "pharmacy/medical supplies."

**Table 1:** Costs^a^ of inpatient treatment, by treatment/service category, for 119 patients with West Nile virus illness, Louisiana, 2002

Treatment/Service category	n^b^	Per patient costs ($)			
		Median	Range	Interquartile range	Total costs ($)
Pharmacy/Medical supplies	115	2,934	16–88,825	994–7,601	887,759
Diagnostic	118	2,417	95–42,064	1,370–4,844	547,935
Room and board	117	1,132	52–16,445	640–2,266	237,917
Medical/Surgical services	75	675	15–21,606	183–3,261	200,233
Intensive care	24	5,526	439–17,769	2,320–11,001	162,360
Rehabilitation^c^	35	425	71–4,202	189–367	32,947
Emergency service	79	271	90–1,416	195–372	25,947
Other	33	109	1–2,620	20–247	8,873
**Total**	119	8,274	623–164,668	3,627–18,197	2,103,971

^a^Economic costs were estimated on the basis of hospital charges, by using Medicare cost-to-charge ratio for Louisiana—0.41 for urban areas and 0.488 for rural areas. ^b^n, number of patients who incurred costs in this category among the 119 patients. Per patient statistics include only the patients who incurred costs in this category. ^c^Rehabilitation costs indicated are those incurred during acute care hospital treatment. Costs of rehabilitation treatment in rehabilitation facilities are not included.

Because charges for healthcare products or services may not represent their true economic cost (Appendix 2), i.e., the opportunity cost of a resource used for producing goods, services, or both (*10**,**11*), we converted hospital charges to economic costs by using Medicare cost-to-charge conversion rates (*12*). Charges made by healthcare providers are generally higher than the cost of resources used (Appendix 2). For Louisiana, the cost-to-charge ratios were 0.410 for urban areas and 0.488 for rural areas (e.g., in urban areas, a $1 charge has an estimated $0.41 economic cost). Two of 12 participating hospitals were in rural areas.

Of 119 patients for whom inpatient treatment charges were available, 7 incurred costs for inpatient rehabilitation treatment. These inpatient rehabilitation treatment charges were provided by acute-care hospital–based rehabilitation centers. Charges were converted into costs as described above. The costs of treatment for the seven patients were then extrapolated to estimate the total costs for all CNS patients requiring rehabilitation by using the methods described earlier.

### Outpatient Costs, Medication, and Durable Medical Equipment

Information for estimating medical doctor visit costs, outpatient rehabilitation treatment costs, and nonmedical costs, including productivity losses, was gathered by interviews using a questionnaire administered by telephone from December 7, 2002, until February 27, 2003 (questionnaire provided in the Online Appendix). Phone numbers for 236 adult patients with nonfatal WNV cases were available from the Louisiana Office of Public Health. Of these 236 persons, 139 were interviewed, 46 did not answer the phone (at least three calls were made at different times of day), 4 were deceased, 2 denied WNV illness, and 16 refused to participate. Twenty-nine of the phone numbers were listed incorrectly or were disconnected.

We collected information about general practice, specialist, and outpatient rehabilitation treatment visits through the patient questionnaire. We estimated the costs for these visits by using a private health insurance claims database (Marketscan database 1999, The MEDSTAT Group, Inc., Ann Arbor, MI). This database is compiled from health insurance claims submitted to 40 self-insured employers and represents over 5 million covered lives across the United States. Average payments made to healthcare providers in the United States in 1999 were calculated for each service. Costs of specialist visits were estimated on the basis of relative prices compared to the national average payments for general practitioners. Relative prices for medical specialists and hospital-based specialists were 1.18 and 3.65 times those of primary care physicians, respectively (*13*). We used the Consumer Price Indices (CPI) for medical care to adjust the 1999 payments for inflation through the year 2002 (*14*).

Charges for outpatient treatment in hospitals were available for 50 patients. Although outpatient treatment costs for hospitalized and nonhospitalized patients might be different, the available data could not be separated and thus the outpatient costs that were estimated based on the combined data for hospitalized and nonhospitalized patients were extrapolated to all reported WNV cases.

Although 60% of the questionnaire respondents indicated outpatient medication expenses, these respondents could not accurately recall the names and amounts of medications taken. Therefore, we did not include outpatient medication costs in the total cost of the outbreak.

The questionnaire was used to gather information about durable medical equipment use. Equipment costs were estimated on the basis of the 1999 MedStat Marketscan database data (Appendix 3) and adjusted to 2002 dollars by using a CPI medical care component. Assuming that the patients for whom durable equipment data were available (139 questionnaire respondents) were representative of all 204 CNS patients, we extrapolated the costs to all CNS patients requiring durable equipment.

### Nonmedical Costs

#### Productivity Losses Attributable to Illness and Death

We used the human-capital method to estimate productivity losses attributable to illness and death (*10*). The productivity losses are measured as income forgone because of illness or premature death. These losses are also referred to as mortality cost. Information about workdays missed by patients or caregivers was obtained through the patient questionnaire. Of 139 respondents, 65 were employed before becoming infected with WNV. Respondents provided information about their earnings; income data were missing for 12 of 65 patients and for 15 of 36 caregivers who missed work to care for a patient. For these cases we used Bureau of Labor Statistics 2001 Louisiana state occupational employment and wage estimates (*15*), converted to 2002 dollars using the ratio of 2002 hourly wages to 2001 hourly wages (*16*). Ten of these 65 patients reported stopping work entirely because of WNV illness. Because the dates when each stopped working were not available and when each could resume work was unknown, we estimated their productivity losses from the second week of August (about half of Louisiana cases occurred before this date) until the last patient interview on February 27, 2003. Respondents provided information on their earnings before they stopped working. We estimated their productivity losses with the methods described.

The Louisiana Office of Public Health provided demographic information about patients who died. For persons<75 years of age, mortality costs were estimated as the present value of labor market earnings and household production based on productivity loss tables (*17*). Because the tables presented the current values of productivity losses at 5-year intervals, we interpolated the present values for consecutive ages within that 5-year interval and chose the values corresponding to deceased patients.

For persons 76–85 years of age, we used productivity loss tables on the annual weighted average earnings (1990 dollars adjusted to 2002 dollars [16]) by age group for labor-force and nonlabor-force persons (*18*). We estimated the expected lifespan for each age group using life tables for the total U.S. population (*19*) (Appendix 4). Then we added the annual earnings by age throughout the expected lifespan of the person, while making adjustments for a 3% discount rate (defined in Appendix 2) to calculate the present value of the person's earnings during future years of his or her lifespan (3% discount rate is recommended by the U.S. Public Health Service Panel on Cost-Effectiveness in Health and Medicine [20]) and a 1% annual productivity increase (1% is the usual assumption for long-term growth in labor productivity [17]).

### Nursing Home, Transportation, and Miscellaneous Costs

Information about nursing home admissions and length of stay because of WNV illness was obtained through the patient questionnaire. We used the average daily cost for nursing homes in Louisiana from General Electric's long-term care insurance data (*21*).

The questionnaire obtained information on the frequency of doctors' visits and the distance that patients had to travel to see a physician. Transportation costs were estimated by using the U.S. federal government reimbursement rate of 36.5 cents per mile (*22*). Information about payments made for home health aides and miscellaneous services, such as cleaning, garden work, or babysitting, was obtained through the questionnaire.

### Costs of Public Agencies

The Louisiana Office of Public Health incurred costs for laboratory support (human serum processing, diagnostic tests), epidemiologic aid (assessment of vector mosquito populations, active surveillance), administrative and clerical activities, and communication services. Information on these expenses was provided by the Louisiana Office of Public Health central office. State and local governments incurred costs for emergency vector control. Only expenditures resulting from the 2002 WNV epidemic in Louisiana that were above and beyond normal operating expenses were attributed to the WNV epidemic.

The core document used to estimate mosquito control program costs in Louisiana was the Louisiana Office of Emergency Preparedness summary of state reimbursement requests by 93 entities, such as mosquito abatement programs, parish police juries (parishes in Louisiana correspond to counties, and police juries to county boards of commissioners or similar local governing bodies in other states), and city governments. Expenses eligible for state reimbursement were for overtime labor, rented equipment, and materials exceeding normal budget expenses from June 1 to mid-August 2002. This amount, however, did not cover "payment-in-kind" activities, such as unpaid overtime, the transfer of employees from one activity to another, and replacement or repair of existing equipment extensively used during the epidemic. Many mosquito control units continued WNV control activities from mid-August until November. After November, we gathered from 18 mosquito control units and local government offices an updated estimate of all expenses incurred in 2002 attributable to the WNV epidemic. The ratio of reimbursement requests to total estimated expenses for these 18 entities was 1:1.7. The total requested state reimbursement amount for the 93 entities was multiplied by this ratio to get an estimate of the total expenses for mosquito control attributable to the WNV epidemic in Louisiana. No data were available to allow us to sample the entities by their size or scope of operation. Therefore, the mosquito control programs and local governments that responded to our inquiries may not have been representative of all the entities.

## Results

The source of data and the estimated number of cases that incurred costs in each cost category are presented in Table 2. A summary of all estimated costs for the 2002 Louisiana WNV epidemic is presented in Table 3. The total estimated cost of the WNV epidemic in Louisiana in 2002 was $20.14 million.

**Table 2:** Source of data and number of West Nile virus (WNV) cases for which data were available and estimated number of all cases that incurred costs in a given cost category, Louisiana, 2002

Cost category	Source	N_available_^a^	N_cost-available_^b^	N_total_^c^	N_cost-total_^d^
Inpatient					
Acute care	12 hospitals with >3 adult patients	119	119	204	204
Rehabilitation facilities	Patient's survey	139	19^e^	204	28
Outpatient					
Hospital care	6 of 12 hospitals	159^f^	50	329	103
Doctors' visits	Patient's survey	139	119	329	282
Rehabilitation facilities	Patient's survey	139	43	204	63
Durable equipment	Patient's survey	139	36	204	54
Productivity losses					
Mortality	Louisiana Office of Public Health data	24	NA^g^	NA	NA
Morbidity	Patient survey	139	99	204	146
Nursing home	Patient survey	139	5	204	7
Transportation	Patient survey	139	139	329	329

^a^N_available_, number of patients for whom data were available from the indicated source, including cases with zero costs. ^b^N_cost-available_, number of patients who incurred costs in that particular category out of all patients for whom data were available. ^c^N_total_, number of patients who potentially could have incurred costs in the given cost category; for example, the number of all WNV cases in Louisiana, or the number of total central nervous system cases in Louisiana. ^d^N_cost-total_ is estimated by the methods described in Appendix 1. ^e^The cost of inpatient rehabilitation treatment was estimated based on seven patients' charges received from four acute-care hospital-based rehabilitation centers. ^f^Of the159 patients for whom hospital charges were available from the 12 acute care hospitals, 50 from 6 hospitals incurred costs for outpatient hospital treatment. ^g^Not applied.

**Table 3:** Summary of costs attributable to 2002 West Nile virus epidemic in Louisiana

Cost category	Cost ($ millions)	% of total epidemic cost
Medical costs		
Acute care	3.60	
Inpatient rehabilitation facility	0.39	
Inpatient treatment subtotal	**3.99**	20
Outpatient hospital care	0.03	
Visits to medical doctors	0.13	
Outpatient rehabilitation facilities, equipment	0.25	
Outpatient treatment subtotal	**0.41**	2
Total medical costs	**4.39**	22
Nonmedical costs		
Mortality	5.40	
Morbidity	1.01	
Productivity losses subtotal	**6.41**	32
Nursing home	0.05	
Transportation, miscellaneous	0.09	
Total nonmedical costs	**6.55**	33
Total cost of illness	***10.94***	***54***
Mosquito control	8.30	
Louisiana Office of Public Health	0.90	
Total public agency cost	***9.20***	***46***
Total cost of epidemic	***20.14***	***100***

### Medical Costs

#### Acute-Care Inpatient Costs and Inpatient Rehabilitation Costs

We received information about acute-care hospital inpatient charges for 119 patients. Total charges for these 119 patients were $5.1 million, from which we estimated an economic cost of $2.1 million (the median cost per patient was $8,274, with a range of $623–$164,688) (Table 1). The economic costs for 71 (60%) patients were <$10,000 (Figure). If we assume that the total number of hospitalized patients with WNV in Louisiana was equal to the number of CNS illness cases, the estimated total costs of inpatient hospitalization were $3.6 million for the 204 CNS illness patients.

**Figure:**
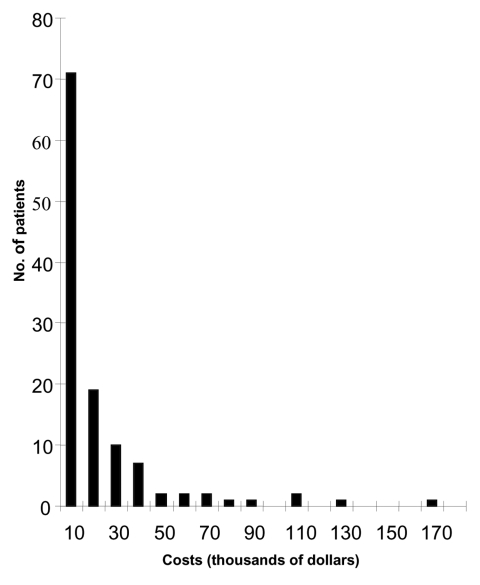
Number of hospitalized patients (N = 119) with West Nile virus infection, by cost of inpatient treatment; Louisiana, 2002.

The median hospital stay was 8 days, with a median of 7 days for intensive care (Table 4). The daily median costs of stay were $160 for a standard room (range $98–$392), $537 for the intensive care unit (range $220–$1226), and $249 for the intermediate, post–intensive care unit (range $161–$314).

**Table 4:** Number of hospital days for 119 patients with West Nile virus illness, Louisiana, 2002

	n^a^	Mean	Median	Range	Interquartile range
Regular room	117	9	7	1–62	4–10
Intensive care	24	11	7	2–29	4–17
Post–intensive care, intermediate intensive care	8	5	3	1–11	2–9
Any room	119	12	8	1–76	4–13

^a^n, number of patients of the 119 patients who incurred costs in a given category.

Nineteen (14%) of 139 survey respondents received inpatient treatment at a rehabilitation facility. Hospital charges were available for seven patients; the total cost for inpatient rehabilitation treatment for those seven patients was $96,556. Using the methods described in Appendix 1, we estimated that 28 of 204 CNS case-patients in Louisiana received inpatient rehabilitation treatment at a total cost of $386,000.

#### Costs of Outpatient Hospital Treatment and Physician Visits

Of 159 patients for whom hospital charges were available, 50 (32%) received outpatient hospital treatment at a total cost of $14,539. Using these numbers, we estimated that 103 of 329 persons reported to the Louisiana Office of Public Health received outpatient hospital treatment, at an estimated cost of $30,000. The estimated total cost of visits to see a primary care doctor, specialist, or both for 139 patients who responded to the survey was $54,572; extrapolating this figure to the 329 reported WNV cases yielded an estimated cost of $129,000.

#### Costs of Outpatient Rehabilitation Therapies and Durable Medical Equipment

Thirty-one (22%) of 139 respondents reported receiving outpatient physical therapy, with an estimated cost of $110,184. Ten of 139 patients reported receiving occupational therapy, with a total estimated cost of $35,207. Two patients received speech therapy, at a total estimated cost of $1,025. The total estimated cost for outpatient rehabilitation therapy for these 139 survey respondents was $146,417; extrapolating this figure to 204 CNS case-patients in Louisiana yielded an estimated cost of $215,000. The cost of durable medical equipment (36 of 139 respondents used medical durable equipment such as a wheelchair, walker, cane, breathing treatment machine, treadmill, and hospital bed) extrapolated to the 204 CNS case-patients was an estimated $31,000.

### Nonmedical Costs

#### Productivity Losses from Illness and Death

For 53 patients who missed work but did not stop working entirely, the estimated productivity losses were $443,000 (the average number of days missed was 50, and the median number of days missed was 37, with a range of 1 to 212 days). Extrapolating this figure to 204 CNS patients, we estimated that 78 missed work, at a total productivity loss of $652,000. For the 10 patients who stopped working entirely, the estimated productivity losses were $157,950. Based on these data, we estimated that 15 of 204 CNS patients stopped working entirely, at a total cost of $237,000 (Appendix 1). Thirty-six of 139 respondents indicated that someone missed work to take care of them; the resulting productivity loss totaled $82,669 dollars. The extrapolated cost for caregivers for the 204 CNS patients was $122,000. The total extrapolated illness cost attributable to WNV infection was $1.01 million.

Twenty-four deaths were attributed to WNV illness in Louisiana in 2002. The median age of deceased patients was 78 (range 27–94). The total estimated mortality cost for these 24 persons was $5.4 million, which was >50% of the illness-associated costs and >25% of the total costs of the epidemic.

#### Nursing Home, Transportation, and Miscellaneous Costs

Five (4%) of 139 patients 45–86 years of age were reported to have spent 21–170 days in a nursing home because of complications from WNV infection. Two of these patients remained in a nursing home at the time of their interviews in December 2002 and February 2003. The estimated total payment for nursing home care for the five patients was $36,956; the total estimated nursing home costs for Louisiana CNS patients were $54,000.

The estimated transportation cost for 139 respondents was $8,354. If one assumes that the transportation costs for the 139 respondents were representative of costs for those who did not participate in our survey, the estimated total cost of transportation for the 329 WNV cases was $20,000.

Twenty survey respondents reported having used home health aides or other services, such as babysitting, house cleaning, or yard work, at a reported total cost of $29,225. When this figure is extrapolated to the 329 WNV cases in Louisiana, miscellaneous expenses were at least $69,000.

### Costs of Public Agencies

#### Mosquito Surveillance and Abatement

From June 1 to mid-August, 2002, a total of 93 public offices requested $4,879,070 as state reimbursement from the Louisiana Office of Emergency Preparedness. Eighteen mosquito control units and local government offices reported their estimated total expenses. Using the ratio of the sums of the requested reimbursement amounts to the total reported expenses of $1:$1.7, we estimated that the cost of mosquito surveillance and abatement programs for these 93 entities was $8.3 million.

### Public Health Office Costs

From June to November 2002, the central state public health office incurred an estimated $886,000 in expenses because of WNV. From this total amount, basic operating expenses cost $586,000, contracts such as for veterinary diagnostic and entomologic services cost $166,000, and laboratory expenses cost $134,000.

## Discussion

We estimated that the costs from June 2002 to February 2003 attributable to the 2002 WNV epidemic in Louisiana were $20.1 million (Table 3). This figure is likely an underestimate since some of the costs associated with illness or public health response were not available, such as costs for outpatient medication and costs incurred by persons with WNV infections who were not identified or reported to Louisiana Office of Public Health. Long-term costs of WNV illness sequelae were not evaluated.

Although the costs of medical care, wages, and cost of living vary by region, we assumed that the Louisiana costs were representative of those elsewhere in order to roughly estimate the magnitude of the WNV epidemic nationwide. Extrapolating to the 4,156 cases (2,942 CNS cases) reported nationwide, the short-term costs of inpatient treatment would be $57.5 million, outpatient treatment costs would be $5.6 million, and nonmedical costs would be $76.7 million, for a sum of $139.8 million. This figure does not include mosquito abatement and prevention costs (mosquito control capabilities vary tremendously from state to state), which accounted for approximately half of the costs in Louisiana.

To our knowledge, only a study of the 1966 St. Louis encephalitis virus epidemic in Dallas, Texas (172 cases, 20 deaths), estimated the cost of a mosquitoborne disease epidemic in the United States (*4*). The total costs of that epidemic were an estimated $796,500 in 1966 dollars. Adjusting each cost component by the appropriate CPI (using CPI for all items or for medical care), the total epidemic cost was $5.4 million in 2002 dollars, from which the largest share was for epidemic control expenditures ($348,500 in 1966 dollars [$1.9 million in 2002 dollars]).

The time frame of our study was from June 2002, when the epidemic was first recognized, until the last date we administered the survey, February 2003, some 3 months after the onset of illness of the last reported case-patient. Several patients, however, likely incurred further costs beyond the date of their interview. Seventy-three (53%) of 139 survey respondents indicated that they expected to get further treatment because of health problems caused by WNV. When, or if, those who stopped working will be able to resume work is also unknown. Another limitation of our study was the possible bias in the estimations that were based on the information gathered by the patient survey. We did not have the information to determine whether differences existed between the nonrespondent and respondent groups.

Although the future incidence of WNV disease cannot be predicted, WNV incidence will likely remain substantially greater than the total incidences of arbovirus infections previously known to be endemic to the United States (*23*). These Louisiana data suggest that even short-term costs attributable to WNV epidemics are substantial.

The costs associated with WNV epidemics such as those documented here can be used to evaluate the economics of WNV prevention and control programs. To fully evaluate the economics of prevention programs, epidemiologic and mosquito control data related to program effectiveness are necessary.

## Appendix 1

### Extrapolation Methods

If we assume that the case-patients for whom data were available were representative of case-patients for whom data were not available, to estimate the total cost of all applicable cases, *C_total_*, first we estimated the total number of cases who would have incurred costs in that particular cost category, *N_cost-total_*._
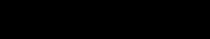
_*N_cost-available_* indicates the number of case-patients who incurred costs in that particular category out of all case-patients for whom data were available, *N_available_*, which also includes case-patients with zero costs. *N_total_* is the number of all case-patients who potentially could have incurred costs in that cost category, for example, the number of total WNV case-patients in Louisiana, or the number of total central nervous system case-patients infected with WNV in Louisiana. The total cost for a given cost category would be:_
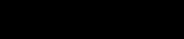
_,where *C_available_* is the cost for available cases.

All extrapolated estimates presented in the paper are rounded to their nearest $1,000.

## Appendix 2

### Explanation of Economic Terms for Noneconomists

#### Economic (True) Cost and Cost-to-Charge Ratios

Economic (true) cost means opportunity cost of a resource. Economists are usually interested in societal costs of health programs—the value of benefits that would have been derived if the resources had been allocated to their next best use, i.e., the opportunity cost of resources. In *perfect markets* (explanation of costs in perfect markets follows the definition of cost-to-charge ratios), the market prices of resources reflect their opportunity costs. Because of healthcare market imperfections (explained below), charges made by healthcare providers do not usually reflect opportunity cost and are generally higher than the cost of resources used (explanation for the reasons of charges being higher than costs in health care is provided in this appendix under subtitle A*symmetric Information*). Large insurance companies and the government (Medicare/Medicaid) reimburse hospitals and physicians at a much lower rate than the charges made by the healthcare provider. These reimbursements are closer to the actual costs of the resources used than the charges made by the healthcare provider.

The common method for estimating the true economic cost of medical services is adjusting the charges through the use of *"cost-to-charge ratios."* Cost-to-charge ratios are coefficients developed by expert panels to convert charges for medical services to their true economic costs. They represent an average estimate of true costs. The Federal Register publishes state by state Medicare cost-to-charge ratios every year. The ratios are different for urban and rural areas.

### Costs in Perfect Markets

Obtaining the opportunity cost of a resource is difficult. In *perfect markets,* the market prices of resources reflect their opportunity costs. Therefore, to determine opportunity costs, we have to collect market prices for goods traded in perfect markets. Perfect market conditions exist when 1) numerous buyers and sellers can enter and withdraw from the market at no cost, 2) all buyers are identical, 3) all buyers possess the same relevant information, and 4) goods and services traded are the same. In reality, one or many conditions of perfect markets are violated in most markets. Economists call them *imperfect markets*. Various methods are used to estimate the costs of resources when conditions for a perfect market are violated or the resources are not traded in the marketplace. Healthcare markets do not meet the conditions for perfect markets for a number of reasons, including those discussed in the following sections.

### Asymmetric Information

Consumers in healthcare markets generally have little information about the treatments medical professionals offer them. They are at a disadvantage to make fully informed choices. Economists refer to such a difference in access to information between market participants as *asymmetric information*. Asymmetric information allows the sellers to charge prices for medical services that are higher than opportunity costs.

#### Market Power

The size and limited number of health insurance companies—the important participants in healthcare markets who "buy" care from providers—gives them considerable market power to influence the prices of goods and services sold in that market. Health insurance companies representing large numbers of subscribers use their weight to negotiate discounts from hospitals and doctors (the "sellers"). Therefore, the prices paid to providers vary with the insurance status of patients and do not correspond to opportunity costs. For more details on economic costs and cost-to-charge ratio method, see Haddix et al. (*24*) or Meltzer (*25*).

### Discount Rate

Discounting is an economic notion that, even in a world of zero inflation, a dollar today would be of higher value to a person than a dollar in the future. A dollar today can be used to purchase a good or service now instead of making the purchase later. This concept is referred to as time preference. The premium placed on benefits today versus the future is reflected in the rate at which a person is willing to exchange present for future costs and benefits. This quantitative measure of time preference is called the *discount rate*. When the costs or benefits under the study continue in the future, in order to make them comparable in terms of the time dimension economists calculate the present value of these costs or benefits by using discount rates. The U.S. Public Health Service Panel on Cost-Effectiveness in Health and Medicine recommends a 3% discount rate for economic studies in health (*26*).

## Appendix 3

### Estimating Costs of Durable Equipment

Costs vary among the types of similar durable equipment. For example, costs for different types of wheelchairs vary considerably. Because the particular type of equipment each patient used could not be accurately ascertained, we estimated the expected cost of that equipment on the basis of data available from the Marketscan database, which provided average national payments for each type of durable equipment. Let *N_i_* be the number of payments reported to Marketscan for the *i^th^* type of the equipment, where *i=1,2, … n*. *N* is the total number of payments reported to Marketscan for all types of that durable equipment:


_
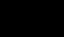
_


*C_i_* is the mean payment for the *i^th^* type of equipment. For certain equipment, such as wheelchair, *C_i_* might represent an average payment for rental of that equipment. Since we do not know the type of equipment the patient bought or rented, we view the relative frequency _
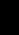
_as the probability of a patient purchasing that particular type of equipment. We estimated the expected cost of the given durable equipment as:


_
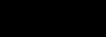
_


## Appendix 4

### Estimating Expected Life Years for a Person

The life table of the total U.S. population for the year 2000 provided numbers of survivors, by 1-year increments, from birth to a given age, starting with a cohort of 100,000 people (*27*). At each age, the expected life years for the surviving cohort was also provided. The expected life years for a person in our study, *ELY_i_,* was estimated as the product of the person's survival rate and the expected life years for the cohort, *ELY_cohort_*, where the survival rate for a person is equal to the ratio of the number of survivors until the expected age for the cohort, *N_survivors_*, to the number of persons in the cohort at a given age, *N_individuals_*:


_
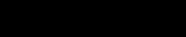
_


The estimated expected life years for a person 76 or 77 years of age were 5 years. For persons 78–81 years of age, the estimated individual life years were 4. For persons 82–84 years, the expected life years were 3. We assumed that for persons >85 years, the productivity losses were 0; therefore, the expected life years for persons >85 were not relevant in our application.

## Online Appendix

### Questionnaire To Assess the Costs of West Nile Virus Illness for Adults

Date when the questionnaire is administered: ----/----/---- (mo/day/year)

(Interviewer to introduce themselves)

Hello. My name is ________, and I am calling on behalf of the Centers for Disease Control and Prevention and the Louisiana Office of Public Health.

We are conducting a survey to determine the costs of the West Nile virus epidemic in Louisiana. This information will help your parish, State of Louisiana, and the Centers for Disease Control and Prevention in planning and strengthening disease control activities, such as mosquito control, to prevent future outbreaks.

I would like to ask you some questions about the expenses you have had because of your West Nile illness.

Participation in this survey is voluntary, and you may stop it at any time or you may choose not to answer any question that you do not care to answer. This survey is anonymous, and your name and other information that could identify you are not recorded on the questionnaire. After the interview nobody will be able to connect you to any of the answers that you give to us. We expect this interview to last about 20 minutes.

(If the patient is unable to participate in the study because of health reasons, ask if someone in the household could answer on the patient's behalf).

NOTE: If the patient is now deceased, mark the box below, and STOP THE INTERVIEW. Mention the following "I am very sorry to hear about the loss. I won't trouble you any further. Thank you for your time."

Patient is now deceased. Mark box ( )—otherwise proceed.

Note to the administrator: Please place an X in the parenthesis ( ) to indicate the answer.

The person answering the questionnaire is the:

1-( ) Patient 2-( ) Patient's spouse 3-( ) Son/Daughter 4-( ) Mother/Father

5-( ) Sister/Brother 6-( ) Friend 7-Other ____________________(please specify)

Gender of patient

( ) Male ( ) Female

Patient's age: (in years)

________

Note: If the patient is 18 years or younger, STOP the interview, explain to the respondent that patients under 18 are not interviewed because of confidentiality reasons. Thank the respondent for their willingness to participate in the study.

### Outpatient Medical Costs

I would like to ask you some questions about your visits to a doctor's office because of your West Nile illness. Please answer them as accurately as possible.

1. Because of your symptoms from the West Nile infection, how many times did you visit a doctor's office? If you have been hospitalized because of West Nile illness, this question is about visits to see a doctor **before hospitalization**.

___________times (Indicate "0" if the patient did not visit a doctor, and "99" if unknown.)

(If "0", skip to question 4.A.)

2. To the best of your recollection, on average, how many minutes did you spend with the doctor on each of these visits? Estimate as best as possible.

______ minutes (Indicate"99" if unknown.)

3. How far, on average, did you have to travel, round trip, to see the doctor? Give the best estimate you can.

______ miles (Indicate "999" if unknown.)

### Complications after Acute Care Hospitalization and Further Treatment

The following questions will be about health complications you may have experienced due to your West Nile illness, and about your further treatment after the hospitalization.

4.A. Did you spend time in a nursing home because of complications caused by West Nile illness?

( ) Yes ( ) No ( ) Don't know ( ) Refuse to answer

(If "No," "Don't know," or "Refuse to answer," skip to question 5.)

4.B. How many days or months in total did you spend in the nursing home because of your West Nile illness? Estimate as best as possible.

____________days (If *still* in the nursing home, indicate the number of days so far and check **here: ( )**.)

5. Because of the symptoms of your WNV illness, did you stay overnight for treatment in a rehabilitation center (other than the nursing home or hospital)?

1- Yes 2- No (SKIP to 6) 9- Don't know (SKIP to 6) 7- Refused (SKIP to 6)

5.A. How many nights in total did you spend in a rehabilitation center? Give the best estimate.

_____________nights (If still in a rehabilitation center, indicate the number of nights so far and check **here: ( )**.)

5.B. From the list I will read to you, indicate the type of treatments that you received during your stay in the rehabilitation center. You may indicate more than one treatment.

1- Physical therapy

2- Speech therapy

3- Occupational therapy

4- Other: please specify ____________________________________

6. Since the first time you were hospitalized, were you hospitalized again for health complications that were related to your West Nile illness?

( ) Yes ( ) No ( ) Don't know ( ) Refuse to answer

(If "No," "Don't know," or "Refuse to answer," skip to question 7.)

6.A. What was the diagnosis for your second hospitalization? (If exact diagnosis is unknown, ask patient/respondent to explain reason for hospitalization as precisely as possible.)

______________________________________________________________________

______________________________________________________________________

6.B. How many nights did you stay in this hospital? Estimate as best as possible.

________________ nights (Indicate 999 if unknown.)

7. Because of your symptoms from West Nile illness, did you visit a physical therapist? If you received physical therapy during your overnight stay in a hospital or rehabilitation center, do not include it here.

( ) Yes ( ) No ( ) Don't know ( ) Refuse to answer

(If "No," "Don't know," or "Refuse to answer," skip to question 8.)

7.A. If "Yes," how many times did you visit a physical therapist after you left the hospital? Estimate as best as possible.

_________ number of times (Indicate "999" if cannot estimate.)

7.B. If "Yes," to the best of your recollection, on average, how many minutes did you spend with the physical therapist on each of these visits? Estimate as best as possible.

_________ minutes (Indicate "999" if cannot estimate.)

7.C. Approximately, how far did you have to travel, round trip, to see the physical therapist? Estimate as best as possible.

______ miles (Indicate "999" if unknown.)

8. Did you visit a speech therapist because of your WN illness? If you received speech therapy during an overnight stay in a hospital or rehabilitation center, do not include it here.

( ) Yes ( ) No ( ) Don't know ( ) Refuse to answer

(If "No," "Don't know," or "Refuse to answer," skip to question 9.)

8.A. If "Yes," how many times did you visit a speech therapist after you left the hospital? If you received speech therapy during an overnight stay in a hospital or rehabilitation center, do not include it here. Estimate as best as possible.

_________ number of times (Indicate "999" if cannot estimate.)

8.B. To the best of your recollection, on average, how many minutes did you spend with the speech therapist on each of these visits? Estimate as best as possible.

_________ minutes (Indicate "999" if cannot estimate.)

8.C. Approximately, how far did you have to travel, round trip, to see the speech therapist? Estimate as best as possible.

______ miles (Indicate "999" if unknown.)

9. Did you receive occupational therapy? If you received occupational therapy during an overnight stay in a hospital or rehabilitation center, do not include it here.

( ) Yes ( ) No ( ) Don't know ( ) Refuse to answer

If "No," "Don't know," or "Refuse to answer," skip to question 10A.

9.A. If "Yes," how many times did you visit an occupational therapist? Estimate as best as possible.

_________ number of times (Indicate "999" if cannot estimate.)

9.B. To the best of your recollection, on average, how many minutes did you spend with the occupational therapist on each of these visits? Estimate as best as possible.

_________ minutes (Indicate "999" if cannot estimate.)

9.C. Approximately, how far did you have to travel, round trip, to see the occupational therapist? Estimate as best as possible.

______ miles (Indicate "999" if unknown.)

10.A. After you left the hospital, did you need equipment or medications, such as nebulizers (a machine that provides respiratory medicines) or home oxygen, to help you with respiratory problems caused by your WNV illness?

( ) Yes ( ) No ( ) Don't know ( ) Refuse to answer

(If "No," "Don't know," or "Refuse to answer," skip to question 11.)

10.B. If "Yes," describe the equipment, type of medication, and amount of the medication or home oxygen that you used to help you with respiratory problems.

___________________________________________________________

___________________________________________________________

11. Since the time you left the hospital, because of your West Nile illness, about how many times have you visited a doctor such as a family doctor, general practitioner, or neurologist? Do not include doctor visits in a nursing home or rehabilitation center. Estimate as best as possible.

____________ number of times (Indicate 0 if no visits, and "99" if unknown.)

(If the answer is 0, skip to the next section, "Productivity losses," read aloud the introduction to that section, then ask question 12.)

11.A. Approximately how far did you have to travel, round trip, to see the doctor? Estimate as best as possible.

______ miles (Indicate "999" if unknown.)

11.B. To the best of your recollection, on average, how many minutes did you spend with the doctor on each of these visits?

______ minutes (Indicate"99" if unknown.)

### Productivity Losses

Now I would like to ask you questions about your job and about the missed workdays because of your West Nile illness.

12. At the time you got sick with West Nile illness, were you working for pay?

( ) Yes ( ) No ( ) Don't know ( ) Refuse to answer

(If "No," "Don't know," or "Refuse to answer," skip to question 24.A.)

13. What was your occupation at the time you got sick with West Nile?

_______________________ (Please specify.)

( ) Don't know

( ) Refused to answer

14. Did you change your occupation, stop working entirely, or take early retirement because of your West Nile illness?

( ) Yes ( ) No ( ) Don't know ( ) Refuse to answer

If "Yes," specify your new occupation. If you took early retirement because of your West Nile illness, indicate "early retirement" as your answer.

______________________________________________________________________

______________________________________________________________________

15. How many days a week did you work during the month before you got sick with West Nile?

____________________ days

( ) Don't know

( ) Refused to answer

16. What was your income rate **from work** (do not include retirement pension, alimony, public assistance, etc.) at the time you got sick with West Nile? Estimate as best as possible. Indicate a payment rate, before taxes, that is easier for you, such as, weekly, monthly, or annual.

$_____________/week (Indicate 99999 if can't estimate.) (Skip to question 18.)

$_____________/month (Indicate 99999 if can't estimate.) (Skip to question 18.)

$_____________/year (Indicate 99999 if can't estimate.) (Skip to question 18.)

( ) Don't know (ASK question 17, then SKIP to question 24.A.)

( ) Refused to answer (ASK question 17, then SKIP to question 24.A.)

17. How many workdays have you missed because of your West Nile illness?

____________________ days (Indicate 999 if unknown.)

(Skip to question 24.A.)

18. Estimate the number of workdays missed due to your West Nile illness while you were paid. (Indicate income rate given in the answer to question 16.)

____________________ days (Indicate "0" if none, and "999" if unknown.)

( ) Don't know

( ) Refuse to answer

19. Has your income rate **from work** changed after your West Nile illness?

( ) Yes

( ) No (Skip to question 24.A.)

( ) Don't know (Skip to question 24.A.)

( ) Refuse to answer (Skip to question 24.A.)

20. If "Yes," is this change in your income due to your West Nile illness?

( ) Yes

( ) No

( ) Don't know

( ) Refuse to answer

21. How many days per week do you work to earn this new income rate?

_________days

(Indicate "0" if none, and "999" if unknown.)

22.A. For how many months have you worked at this new income rate?

________months

22.B. What is this new income rate (do not consider the income from retirement pension, alimony, public assistance, etc.)? Estimate as best as possible. Choose a rate before taxes that is easier for you, such as weekly, monthly, or yearly.

$_____________/week (Indicate 9999 if can't estimate.)

$_____________/month (Indicate 99999 if can't estimate.)

$_____________/year (Indicate 99999 if can't estimate.)

( ) Don't know

( ) Refuse to answer

(If the patient stopped working, indicate "0" and skip to question 24.A.)

23. How many workdays have you missed due to health reasons related to your West Nile illness during the period while earning this new income rate?

____________________ days (Indicate "0" if none, and "999" if unknown.)

24.A. Did someone else miss work to take care of you because of your West Nile illness?

( ) Yes ( ) No ( ) Don't know ( ) Refuse to answer

(If "No," "Don't know," or "Refuse to answer," skip to section Miscellaneous Services and Homecare, read aloud the introduction to that section, and proceed to question 28.A.)

24.B If "Yes," estimate the total number of workdays this person missed to take care of you when you were sick with West Nile. If there were more than one person, consider only the workdays missed by the one who took care of you most.

__________ days ( Indicate 999 if unknown).

25. How many days a week did this person work right before he or she started to take care of you while you were sick with West Nile?

____________days (Indicate "9"[? Not 999?] if unknown.)

26. What was the occupation of the person who took care of you most when you were sick with West Nile? _________________________________

27. From the list I will read to you now, please specify one of the income categories that best describes the average annual income, before taxes, for the person who took care of you while you were sick with West Nile.

1) $0

2) $1–$20,000

3) $20,001–$30,000

4) $30,001–$40,000

5) $ 40,001–$50,000

6) $ 60,001–$70,000

7) $ 70,001–$100,000

8) $100,000 or greater

9) Unknown

### Miscellaneous Services and Homecare

Now I would like to identify any other expenses you may have had due to your West Nile illness. Please answer the questions as accurately as possible.

28.A. Did you use home health aide services, such as help with bathing or getting dressed, because of your West Nile illness?

( ) Yes ( ) No ( ) Don't know ( ) Refuse to answer

28.B. If "Yes," how much did you pay for the home health aide services? Give the best estimate.

______________ total $ (Indicate 9999 if unknown.)

29.A. Was your home modified to accommodate you because of this illness?

( ) Yes ( ) No ( ) Don't know ( ) Refuse to answer

29.B. If yes, how much did you pay to modify your home to accommodate you?

___________ total $ (Indicate 9999 if unknown.)

30. Did you use any miscellaneous services such as babysitting, house cleaning, and transportation due to your West Nile illness?

( ) Yes ( ) No ( ) Don't know ( ) Refuse to answer

30.A. If yes, please specify which services you used? _________________________

30.B. If "Yes," how much did you pay for these services?

_____________________total $ (Indicate 9999 if unknown.)

31. Please list any expenses you have had because of your West Nile illness that we have not asked about. Indicate the type of that expenditure and the total amount.

____________(total $) for _________________________________________

____________(total $) for _________________________________________

32A. Do you have any type of health insurance coverage, including Medicare or Medicaid?

1- Yes 2- No(Skip to 33) 9- Don't Know(Skip to 33) 7- Refused(Skip to 33)

32.B. If yes, from the list I will read to you, indicate the type of health insurance that covered for the treatment or other expenses you had because of your WNV illness. You can indicate more than one type of insurance.

1- Medicare

2- Medicaid

3- Health Insurance provided by employer

4- Other. Please specify ____________________________________________

33. During the next 6 months, do you expect to get further treatment, such as physical therapy, speech therapy, or visiting a doctor because of any health problems caused by your West Nile illness?

( ) Yes ( ) No ( ) Don't know ( ) Refuse to answer

34. During the next 6 months, do you think you will need to go to a nursing home because of health complications caused by your West Nile illness?

( ) Yes ( ) No ( ) Don't know ( ) Refuse to answer

### Public Assistance and Disability Payments

Now I would like to ask you questions about public assistance and disability payments that you may be receiving, or expect to receive in the near future, connected with your West Nile illness.

35.A. Do you now receive any type of public assistance because of your West Nile illness (money from the state or federal government, social security, etc)?

( ) Yes ( ) No ( ) Don't know ( ) Refuse to answer

(If "No," "Don't know," or "Refuse to answer," skip to question 36.A.)

35.B. If "Yes," list the names/types of the public assistance. Indicate the annual or monthly amount of the assistance, and the date when you started receiving it [enter the information in the table below].

**Table:** 

Name/type of public assistance	Annual amount, $/year	Monthly amount, $/month	Month/year assistance started
			
			
			

**Table:** 

36.A. Have you applied for any public assistance (which you are not yet receiving) because of health complications caused by your West Nile illness (money from state, or federal government, social security, etc.)?


( ) Yes ( ) No ( ) Don't know ( ) Refuse to answer

36.B. If *yes*, what are the names of the public assistance, the annual amount of the assistance from each source, and the date (month and year) when you will start receiving it? If you are not sure about the amount of the assistance, or the date you will start receiving it, give the best estimate.

**Table:** 

Name of public assistance	Expected annual amount, $/year	Expected monthly amount, $/month	Month/year assistance is expected to start
			
			
			

**Close interview:** "Thank you sir/ma'am for your time and patience in helping us by answering our questions. I wish to repeat that your answers were recorded in an anonymous manner, and nobody will be able to connect you to any of the answers.

Do you have any questions about today's interview? [Interviewer should answer any direct questions. Anything that you don't know, encourage the respondent to contact us: Dr. Armineh Zohrabian, Centers for Disease Control and Prevention (CDC), phone: 970-266-3553, or Dr. Martin Meltzer, CDC, phone: 404-371-5353.]"
